# A comparative study of *R* functions for clustered data analysis

**DOI:** 10.1186/s13063-021-05900-7

**Published:** 2021-12-27

**Authors:** Wei Wang, Michael O. Harhay

**Affiliations:** 1 Clinical Trials Methods and Outcomes Lab, Palliative and Advanced Illness Research (PAIR) Center, Perelman School of Medicine, University of Pennsylvania, Philadelphia, PA USA; 2 Department of Biostatistics, Epidemiology and Informatics, Perelman School of Medicine, University of Pennsylvania, Philadelphia, PA USA

**Keywords:** Clustered data analysis, Cluster randomized trials, Generalized estimating equations, Mixed effects models

## Abstract

**Background:**

Clustered or correlated outcome data is common in medical research studies, such as the analysis of national or international disease registries, or cluster-randomized trials, where groups of trial participants, instead of each trial participant, are randomized to interventions. Within-group correlation in studies with clustered data requires the use of specific statistical methods, such as generalized estimating equations and mixed-effects models, to account for this correlation and support unbiased statistical inference.

**Methods:**

We compare different approaches to estimating generalized estimating equations and mixed effects models for a continuous outcome in R through a simulation study and a data example. The methods are implemented through four popular functions of the statistical software *R*, “geese”, “gls”, “lme”, and “lmer”. In the simulation study, we compare the mean squared error of estimating all the model parameters and compare the coverage proportion of the 95% confidence intervals. In the data analysis, we compare estimation of the intervention effect and the intra-class correlation.

**Results:**

In the simulation study, the function “lme” takes the least computation time. There is no difference in the mean squared error of the four functions. The “lmer” function provides better coverage of the fixed effects when the number of clusters is small as 10. The function “gls” produces close to nominal scale confidence intervals of the intra-class correlation. In the data analysis and the “gls” function yields a positive estimate of the intra-class correlation while the “geese” function gives a negative estimate. Neither of the confidence intervals contains the value zero.

**Conclusions:**

The “gls” function efficiently produces an estimate of the intra-class correlation with a confidence interval. When the within-group correlation is as high as 0.5, the confidence interval is not always obtainable.

**Supplementary Information:**

The online version contains supplementary material available at (10.1186/s13063-021-05900-7).

## Introduction

### Clustered data

Clustered data arise when the study population can be classified into different groups (referred to as clusters), and the measurements of subjects, in particular the response, within the same cluster are more alike than those in other clusters. For instance, in cluster-randomized trials, entire groups of participants such as classrooms, clinics, communities, or hospitals, rather than individuals, are randomly assigned to intervention arms [1, 2].

The key feature of clustered data is that the similarity (or homogeneity) of measurements within the same cluster induces a correlation. That is, measurements within a cluster are likely to be correlated, whereas those from separate clusters are regarded as independent. The intra-class correlation coefficient (ICC) measures this similarity of the responses within a cluster and can be defined as a function of the variance components in the model: variation between clusters and within clusters [3, 4]. Since the responses within a cluster do not contribute completely independent information, the “effective” sample size is less than the total number of subjects from all clusters [5–7].

### Statistical methods

Classical statistical methods such as ordinary least squares regression assume that each individuals’ data is independent. The clustered data have a hierarchical structure where individuals are not likely independent within the same cluster (i.e., ICC > 0). Thus, methods taking the correlation into account, such as generalized estimating equation (GEE) and mixed-effects models, are well suited for the analysis of clustered data [8–10].

GEE models can be viewed as an extension of generalized linear models for correlated data where a within-cluster correlation structure is specified [11]. Parameter estimates are then obtained as solutions of the estimating equations [12]. In mixed-effects models, the cluster effect is a random variable representing a random deviation for a given cluster from the overall fixed effects [13–15]. Maximum likelihood estimation is often used to obtain estimates of parameters via iterative algorithms such as the expectation-maximization (EM) algorithm and the Newton-Raphson algorithm [16–21]. As shown in [22], for normal outcomes, GEE reduces to the score equation of the maximum likelihood estimation only when there are no missing observations and the correlation is unstructured. A further comparison shows that GEE and mixed-effects models produce the same generalized least squares estimator of the fixed effects [23]. We will review the derivation of the fixed effects estimator in the next section.

Simulation studies have been conducted to compare the two methods for analyzing continuous outcomes with an emphasis on the fixed effects components. In the comparison of the estimation and the coverage probability of the confidence intervals, Park [22] found that the GEE estimation was more sensitive to missing observations. In the study [24], the authors compared the estimation and the nominal level of hypothesis testing and made several recommendations. For instance, if knowledge is available to specify the covariance structure correctly, the maximum likelihood estimation is slightly more efficient for balanced or near balanced data. When there is concern about the misspecification of the covariance structure, GEE is preferred when the number of clusters is larger than 20. For hypothesis testing, Kahan et al. [25] and Leyrat et al. [26] found that without an appropriate correction, both methods can lead to inflated type I error rates (finding a statistically significant treatment effect when it does not exist) when the number of clusters is smaller than 40. *R*, *SAS*, and *Stata* commands to correct the type I error rate are provided in [26].

### *R* functions

In this study, we compared the performance of the GEE method and the linear mixed-effects model to analyze clustered data through the implementation of both popular and newer packages of the statistical software *R* [27]. Specifically, the “geese” function of the *geepack* package (1.3.2) fits a GEE model [28, 29]. The “gls” function of the *nlme* package (3.1.149) [30, 31] fits a linear model using generalized least squares where the errors are allowed to be correlated. Two frequently used functions for conducting linear mixed-effects model analysis are “lme” of the *nlme* package [30, 31] and “lmer” of the *lme4* package [32] (1.1.25). Detailed implementation of these functions is provided in the “Implementation” subsection of the next section and also summarized in Table 1.

**Table 1:** Summary of how to obtain the CI’s of the fixed effects and the variance-covariance parameters

	95% confidence intervals of parameters		
	***β***	{*σ*^2^,*ρ*}	{*σ*_*u*_,*σ*_*ε*_}
geese	Estimate ± 1.96 standard deviation		–
gls	confint	intervals(,which=“var-cov”)*	–
	intervals(,which=“coef”)		
lme	intervals(,which=“fixed”)	–	intervals(,which=“var-cov”)
lmer	confint(,method=“Wald”)	–	confint(,method=“profile”)
	confint(,method=“profile”)		

^*^Applying the “intervals” function to a “gls” fitted object returns the CI of *σ* instead of *σ*^2^

## Methods

We compared the performance of the four functions via a simulation study and through a real data example. In the simulation study, we compared the computation time; the mean squared error (MSE) of estimating all the model parameters, including the ICC; and the coverage proportion of the 95% confidence intervals. Parameter estimates in the linear mixed-effects models are found by maximizing the likelihood of the data. In the following, we review the model setup followed by the simulation study and the example dataset.

### Model review

Let *n*_*i*_ be the number of subjects in the *i*-th cluster and let **1**_*i*_ be a *n*_*i*_×1 vector of one’s. **I**_*i*_ is a square identity matrix of dimension *n*_*i*_ and let **J**_*i*_ be a square matrix of one’s of dimension *n*_*i*_. Let *u*_*i*_ be the random effect associated with the *i*-th cluster. The linear mixed-effects modeling of the *i*-th cluster’s response **y**_*i*_ is

$${} \mathbf{y}_{i}=\mathbf{X}_{i}{\boldsymbol{{\beta}}}+\mathbf{1}_{i} u_{i}+{\boldsymbol{\epsilon}}_{i}, \ \ \ u_{i}\sim \mathrm{N}\left(0,\sigma^{2}_{u}\right), \ \ {\boldsymbol{\epsilon}}_{i}\sim \mathrm{N}\left(\mathbf{0},\sigma_{\epsilon}^{2}\mathbf{I}_{i}\right).$$

The matrix **X**_*i*_ is the design matrix and ***β*** is the unknown fixed effects. The random vector ***ε***_*i*_ represents the error and is independent of *u*_*i*_. Independence is also assumed between *u*_*i*_ and *u*_*j*_, and between ***ε***_*i*_ and ***ε***_*j*_ whenever *i*≠*j*. It follows that **y**_*i*_∼N(**X**_*i*_***β***,***Σ***_*i*_), where ${\boldsymbol {\Sigma }}_{i}=\sigma ^{2}_{u}\mathbf {J}_{i}+\sigma _{\epsilon }^{2}\mathbf {I}_{i}$. Let elements of **y**_*i*_ be *y*_*ij*_, *j*=1,…,*n*_*i*_. We obtain $\text {Var}(y_{ij})=\sigma ^{2}_{u}+\sigma _{\epsilon }^{2}$ and $\text {Cov}(y_{ij},y_{ik})=\sigma ^{2}_{u}$ where *j*≠*k*. The intra-class correlation coefficient *ρ* is naturally defined by the variance components $\sigma ^{2}_{u}$ and $\sigma _{\epsilon }^{2}$ as

1 $$ \rho\equiv \text{Corr}(y_{ij},y_{ik})= \frac{\sigma^{2}_u}{\sigma^{2}},\ \text{where}\ \sigma^{2}=\sigma^{2}_{u}+\sigma_{\epsilon}^{2}.  $$

By definition, 0<*ρ*<1 since both $\sigma ^{2}_{u}$ and $\sigma _{\epsilon }^{2}$ are positive.

The above modeling uses the random effect *u*_*i*_ explicitly to explain within cluster correlation. From a marginal model perspective, one can instead start with the modeling **y**_*i*_∼N(**X**_*i*_***β***,***Σ***_*i*_) directly with a special structure for ***Σ***_*i*_. Let *ρ*=Corr(*y*_*ij*_,*y*_*ik*_) and *σ*^2^=Var(*y*_*ij*_), and sequently we get ***Σ***_*i*_=*σ*^2^[**I**_*i*_+*ρ*(**J**_*i*_−**I**_*i*_)]. Using this marginal parametrization {*σ*^2^,*ρ*}, the matrix ***Σ***_*i*_ is positive definite if − 1/(*n*_*i*_−1)<*ρ*<1 [30, 33–35]. That is, *ρ* does not have to be positive as it was defined from a variance components perspective. At the same time, we also observe that when *n*_*i*_ is large, the boundary − 1/(*n*_*i*_−1) can be very close to 0. Starting with this parameterization, we derive the corresponding relationship of (1) as

2 $$ \sigma^{2}_{u}=\sigma^{2}\rho,\ \ \sigma_{\epsilon}^{2}=\sigma^{2}(1-\rho).   $$

It is clear that ${\boldsymbol {\Sigma }}_{i}^{-1}=\{\mathbf {I}_{i}-\rho \mathbf {J}_{i}/[1+(n_{i}-1)\rho ]\}/\sigma ^{2}(1-\rho)$. Let $\mathbf {T}_{i}(n_{i},\rho) \equiv \mathbf {T}_{i}(\rho)=\mathbf {I}_{i}-\rho \mathbf {J}_{i}/[1+(n_{i}-1)\rho ]=\sigma ^{2}(1-\rho){\boldsymbol {\Sigma }}_{i}^{-1}$. The estimate of ***β*** follows as $\hat {{\boldsymbol {\beta }}}=\left \{ \sum _{i=1}^{n_c} \mathbf {X}_{i}^{\prime } {\boldsymbol {\Sigma }}_{i}^{-1}\mathbf {X}_{i}\right \}^{-1}\left \{\sum _{i=1}^{n_{c}}\mathbf {X}_{i}^{\prime } {\boldsymbol {\Sigma }}_{i}^{-1}\mathbf {y}_{i}\right \}$ which reduces to

$$\hat{{\boldsymbol{\beta}}}=\left\{ \sum\limits_{i=1}^{n_c} \mathbf{X}_{i}^{\prime}\mathbf{T}_{i}(\rho)\mathbf{X}_{i}\right\}^{-1}\left\{\sum\limits_{i=1}^{n_{c}}\mathbf{X}_{i}^{\prime} \mathbf{T}_{i}(\rho)\mathbf{y}_{i}\right\}. $$

The variance-covariance matrix of the estimate has the form

$$\text{Cov}(\hat{{\boldsymbol{\beta}}})\,=\,\!\left\{\! \sum\limits_{i=1}^{n_c} \mathbf{X}_{i}^{\prime} {\boldsymbol{\Sigma}}_{i}^{-1}\mathbf{X}_{i}\!\right\}^{-1}\!\,=\,\sigma^{2}(1-\rho)\!\left\{ \sum\limits_{i=1}^{n_{c}} \mathbf{X}_{i}^{\prime} \mathbf{T}_{i}(\rho)\mathbf{X}_{i}\!\right\}^{-1}. $$

We notice that if there is no within cluster correlation, i.e. *ρ*=0, then **T**_*i*_(0)=**I**_*i*_ and $\hat {{\boldsymbol {\beta }}}$ is simply the ordinary least squares model. In the extreme case of perfect correlation, i.e., *ρ*=1, then **T**_*i*_(1)=**I**_*i*_−**J**_*i*_/*n*_*i*_ and we get

$$\begin{array}{@{}rcl@{}} \hat{{\boldsymbol{\beta}}}|_{\rho=1}&=&\left[ \sum\limits_{i=1}^{n_c} \mathbf{X}_{i}^{\prime}\mathbf{T}_{i}(1)\mathbf{X}_{i}\right]^{-1}\left[\sum\limits_{i=1}^{n_c}\mathbf{X}_{i}^{\prime} \left(\mathbf{y}_{i}-\mathbf{1}_{i}\bar{y}_{i}\right)\right]. \end{array} $$

In this scenario, *y*_*ij*_=*y*_*ik*_ and $\mathbf {y}_{i}=\bar {y}_{i}$, so $\hat {{\boldsymbol {\beta }}}=\mathbf {0}$.

### Simulation

Our simulation setups are similar to those in the literature. In the simulation, Feng et al. [24] tried the number of clusters *n*_*c*_=10, 20, and 50 with cluster sizes 10, 30, and 100, and *ρ*=0.1, 0.5. The simulation study [25] considered two scenarios of 5 patients per cluster with *ρ*=0.15, and of 100 patients per cluster with *ρ*=0.01. The number of clusters *n*_*c*_ varied from 6, 10, 20, …, 90, and 100. The simulation scenarios in [26] include *ρ*=0.001, 0.01, and 0.05, and *n*_*c*_=4, 6, 8, 10, 20, 30, 40, and 200. The average cluster size ranges from 7 to 300. A review of published cluster-randomized trials by Kahan et al. [25] shows that the median number of clusters was 25 with interquartile range 15 to 44, 14% of the trials had fewer than 10 clusters, and 9% of the trials had more than 100 clusters. The cluster size had a median of 31 and an interquartile range 14 to 94.

In our study, we tried *n*_*c*_=10, 30, 50, and 100. In each cluster, the number of subjects was simulated from a normal distribution after rounding with mean *m*=50 and 100, and standard deviation 5. The sample size calculation in [36] preassumed an ICC of 0.05. Some studies also found large ICC values such as 0.47 with 95% confidence interval [0.29,0.65] [6, Table I] and 0.60 [37, Table 4.4.2]. In our setup, we considered *ρ*=0.05, 0.1, and 0.5. We simulated two covariates independently from a Bernoulli distribution with a probability of success of 0.5, and from a standard Normal distribution. The associated regression coefficients are respectively *β*_1_=−2, and *β*_2_=1.5, and the intercept in the regression model is *β*_0_=1. We simulated the outcome from a marginal model with the variance parameter *σ*^2^=0.6. In each of the settings, we examined 2000 simulations.

### Example dataset

We reanalyzed data from the cluster-randomized controlled trial in [36]. In this study, participants with hypertension from 15 clusters in rural India were recruited and randomized to the intervention or usual care in a 1:2 ratio. The study hypothesis was that a CHW (community health worker)-led group-based education and monitoring intervention would result in improved blood pressure control. Outcomes were assessed approximately two months after completion of the intervention.

One of the main outcomes in this trial was the change in diastolic blood pressure (DBP), defined as follows: the DBP at baseline minus the DBP at follow-up. Fixed effects in the analysis include the following variables: age, sex, diastolic blood pressure at baseline (mm Hg), education, use of antihypertensive medications, change in BMI (body mass index) defined as BMI at follow-up minus the BMI at baseline, number of serves of fruit per week at follow-up, and self-reported drinking alcohol at least once in the 30 days prior to follow-up. The education variable has four categories: no formal schooling, class 1 to 6, class 7 to 11, and class 12 or more.

**Fig. 1:**
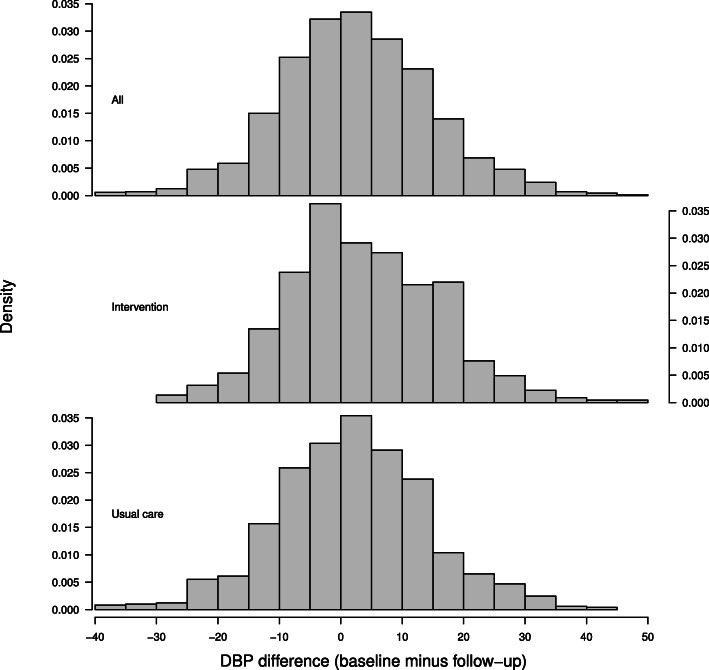
Histogram of DBP difference by groups

**Fig. 2:**
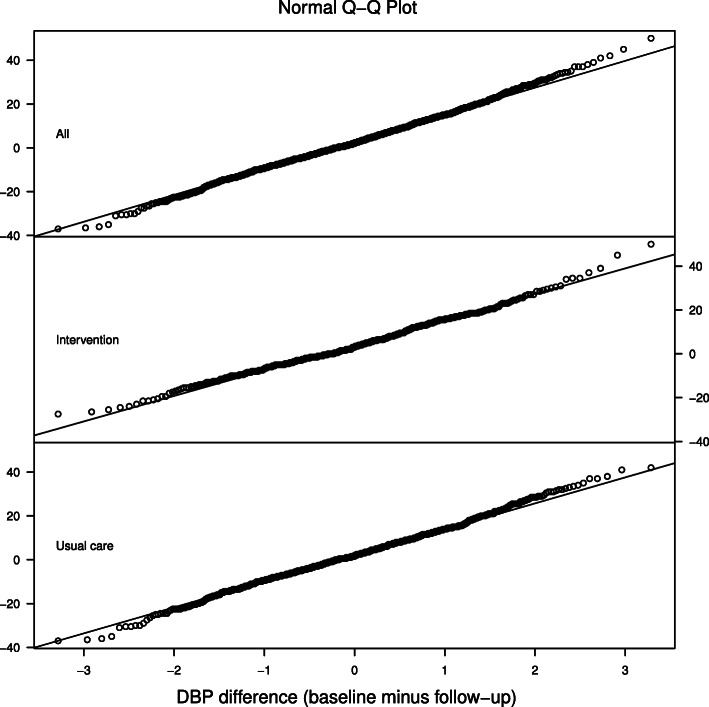
Normal Q-Q plot of DBP difference by groups

We analyzed the data from the 1428 participants with no missing values. The histogram of the outcome shows a bell shaped pattern (Fig. 1). The intervention group has a larger proportion of a positive difference than the usual care group suggesting more DBP decline at the follow-up. The normal quantile-quantile plot in Fig. 2 shows the normal distribution assumption of the outcome is plausible which provides a justification of the application of linear mixed-effects models.

### Implementation

All the four *R* functions compute $\hat {{\boldsymbol {\beta }}}$ and the corresponding confidence interval (CI), but they adopt different parameterizations for the variance-covariance matrix. The function “geese” uses {*σ*^2^,*ρ*} though a specification of an “exchangeable” correlation structure. The function “gls” uses a compound symmetry structure of the parameters {*σ*,*ρ*}. Both “lme” and “lmer” find estimates and CI’s of {*σ*_*u*_,*σ*_*ε*_}. It is straightforward to obtain estimates of *σ*^2^, or $\sigma ^{2}_{u}$ and $\sigma _{\epsilon }^{2}$ from their corresponding square root estimates. We can then find estimates of another parameterization using Eqs. (1) or (2). As the “geese” method does not fit in the framework of hierarchical modeling with random effects, it is not appropriate to find its estimates of $\{\sigma ^{2}_{u},\sigma _{\epsilon }^{2}\}$ using Eq. (2) due to a possible negative $\hat {\rho }$. Thus, we do not include it for the comparison of estimating {*σ*_*u*_,*σ*_*ε*_}. Methods obtaining the CI’s of *ρ* have been discussed in [38, 39]. In the model-based setup, the CI of *ρ* is readily available from the output of the “geese” or “gls” fitted object. Below we explain how to get the CI’s of the model parameters with the summary presented in Table 1.

From the “geese” output, we apply the estimate ± 1.96 standard deviation rule to obtain the CI’s. We apply two generic functions “confint” and “intervals” to “gls”, “lme”, or “lmer” fitted objects. The “confint” function assumes normality and has two options. The option *method=“Wald”* returns approximate CI’s of the fixed effects based on the estimated local curvature of the likelihood surface. The other option *method=“profile”* computes a likelihood profile and find the appropriate cutoffs based on the likelihood ratio test [32]. The “intervals” function calculates approximate confidence intervals for the parameters in the linear model using a normal approximation to the distribution of the maximum likelihood estimators. The estimators are assumed to have a normal distribution centered at the true parameter values and with covariance matrix equal to the negative inverse Hessian matrix of the log-likelihood evaluated at the estimated parameters [30, 31].

## Results

### Simulation

With detailed comparison results presented in the supplementary material, we summarize our findings in the following text. The “lme” approach took the least computation time, 1.4 h, followed by the “gls” approach, 2.41 h (Table S1). Sometimes the “gls” approach failed to construct the confidence intervals for the variance-covariance parameters when *ρ*=0.5 (Table S2). The number of failure increases with the number of clusters *n*_*c*_ and also the cluster size *m*. When *n*_*c*_=100 and *m*=100, there were 528 failures out of 2000 simulations. Occasionally, the “lme” and “lmer” approaches also failed to construct the confidence intervals. The performance of the four approaches of estimating the model parameters is very similar with almost identical standard deviation and MSE (Tables S3-S6).

Next we summarize the performance of the different functions on the coverage proportion of the model parameters. First, in general, the coverage proportions of the fixed effects are very similar among “gls”, “lme”, and “lmer” approaches (Tables S7-S9). They are very close to the nominal 95% level except for *β*_0_ when *n*_*c*_=10 (Table S7). In that case, confidence intervals obtained by specifying the “profile” option of the “confint” function to a “lmer” fitted object outperforms the others. Their coverage proportions are generally closer to the nominal 95% level than the “geese” approach when *n*_*c*_ is less than 100. Second, though the coverage proportions of {*σ*^2^,*ρ*} of “geese” or {*σ*,*ρ*} of “gls” are usually below the 95% nominal level, the “gls” method generally provided better coverage (Table S10). Third, both the “lme” and “lmer” produced coverage proportions about 95% for *σ*_*ε*_, and for *σ*_*u*_ when *n*_*c*_≠10. When *n*_*c*_=10, we observed unstable performance of over coverage or under coverage (Table S11).

### Example dataset

All the four methods produced similar results that suggested more DBP reduction in the intervention group, 2.161 mm Hg by “geese” and 2.252 by the other three methods (Table 2). The 95% confidence interval bounds of the intervention effect are slightly different. However, our conclusion is consistent with the finding in [36] where the analysis was conducted in Stata (Stata IC/11.2, StataCorp, College Station, TX, USA). DBP declined 2.1 mm Hg more in the intervention group with a 95% confidence interval of (0.6,3.6), and the estimation of ICC was 0.02. The “gls” method gives a positive $\hat {\rho }$ and a confidence interval does not contain 0. The “geese” method produces a negative $\hat {\rho }$ with negative confidence interval bounds.

**Table 2:** CI’s of the fixed effects and the variance-covariance parameters

	Intervention effect	*ρ*	*σ*^2^ or *σ*	*σ*_*u*_	*σ*_*ε*_
geese	2.161 (1.060, 3.263)	-0.076 (-0.125, -0.027)	101.768 (93.518, 110.019)		
gls	2.252 (0.597, 3.907)	0.012 (3.7×10^−4^, 0.405)	10.093 (9.727, 10.473)	1.107	10.032
	2.252 (0.595, 3.908)				
lme	2.252 (0.602, 3.901)	0.012	10.093	1.107 (0.589, 2.081)	10.032 (9.669, 10.409)
lmer	2.252 (0.604, 3.900)	0.012	10.093	1.107 (0.234, 2.111)	10.032 (9.673, 10.414)
	2.252 (0.525, 4.086)				

The “geese” method finds the point estimate and CI of the parameter *σ*^2^ instead of *σ* as the other methods. The order of the CI’s of the intervention follows the order of the CI’s of *β* in Table 1

## Discussion and conclusion

Throughout our study, we compare the performance of the four *R* functions, “geese”, “gls”, “lme”, and “lmer”, of analyzing single level clustered data. The “exchangeable” correlation structure of the “geese” function and the compound symmetry structure of the function “gls” both provide a single-level cluster model. We note that the “lme” and the “lmer” function can model multi-level data, and the “lmer” function is capable of modeling crossed random effects. The *lme4* package also includes generalized linear mixed model capability via the “glmer” function. It does not currently implement *nlme*’s features for modeling heteroscedasticity of residuals or offer the same flexibility for composing complex variance-covariance structures.

Our simulation study found that all four methods perform equally well for model parameters estimation. This result is consistent with the study in [24]. It was found that the MSEs are very similar except when the number of clusters is 10 where the linear mixed-effects model method has slightly smaller MSE than the GEE method using SAS PROC MIXED. We observe similar coverage proportions of the fixed effects among “gls”, “lme,” and “lmer” approaches. They are generally closer to the nominal 95% level than the “geese” approach when the number of clusters is less than 100.

The estimated ICC from the “geese” method can be negative, and the confidence interval of the ICC from the “gls” method provides better coverage. However, when the ICC is large as *ρ*=0.5, confidence intervals is not always obtainable from the “gls” method. In our comparison of the coverage of the variance-covariance parameters in the model, the “lme” and the “lmer” methods have similar performance while the former is considerably faster. The latter provides better coverage of the intercept in the model when the number of clusters is 10.

In the simulation settings, we examined that the “gls” function is preferable to analyze single-level clustered data. The limitations of our simulation study include the lack of the scenario of the very large number of clusters (e.g., 200 as in [26]) or the scenario of small ICC values (e.g., 0.01 as in [25, 26]). It may also be of interest to further compare the performance of the four functions for complex trials such as stepped-wedge cluster-randomized trials. In a stepped-wedge cluster-randomized trial, all clusters begin in the control phase and then are randomized to interventions at different time points [40–43]. Simulations have been conducted to investigate the effect of varying degrees of imbalance in cluster size on the power [44].

## Supplementary Information

**Additional file 1** The supplementary material in a pdf format contains the tables of the simulation study results.

## Data Availability

The data analyzed in our example were originally studied in [36]. The data file and the data dictionary are publicly available from the figshare database (accession number 10.26180/5cc80de987113). The data were reanalyzed without collaboration with the original study authors.

## Abbreviations

BMI: Body mass index
CHW: Community health worker
CI: Confidence interval
DBP: Diastolic blood pressure
EM: Expectation-maximization
GEE: Generalized estimating equation
ICC: Intra-class correlation coefficient
MSE: Mean squared error
