# Supplementary material for: A comparative study of R functions for clustered data analysis
Source: Trials. 2021 Dec 27;22:959. doi: 10.1186/s13063-021-05900-7 (PMC8711156; doi:10.1186/s13063-021-05900-7)
Supplement: Supplementary file 1 — Additional file 1 The supplementary material in a pdf format contains the tables of the simulation study results. [file 13063_2021_5900_MOESM1_ESM.pdf]

RESEARCH

Supplementary Material of
A comparative study of *R* functions for clustered data analysis

Wei Wang[1*]

and Michael O. Harhay[1,2]

*Correspondence:
wei.wang@pennmedicine.upenn.edu
[1]Clinical Trials Methods and Outcomes Lab, Palliative and Advanced Illness Research (PAIR) Center, Perelman School of Medicine, University of Pennsylvania, Philadelphia, PA, USA
Full list of author information is available at the end of the article

Table S1 Computation time (hours) of the simulations on a Windows 10 enterprise platform with a 16 GB RAM and an Intel(R) Core(TM) i7-8700 CPU @ 3.20 GHZ processor. The columns correspond to the total user time, the system CPU time, and the “real” elapsed time since the process was started.

	User	System	Elapsed
gee	8.14	0.04	8.33
gls	1.63	0.65	2.41
lme	1.27	0.08	1.40
lmer	11.14	0.14	11.35

Table S2 Number of the failures of obtaining the 95% confidence intervals of the variance-covariance parameters out of the 2000 simulations. *The fitting algorithm also failed to produce parameter estimates.

		m=50			m=100		
Approach	n_c	$\rho = 0.05$	$\rho = 0.1$	$\rho = 0.5$	$\rho = 0.05$	$\rho = 0.1$	$\rho = 0.5$
gls	10	–	–	44	–	–	88
	30	–	–	131	–	–	225
	50	–	–	175	–	–	358
	100	–	–	338	–	–	528
lme	10	20	3	–	11	–	–
	100	–	–	–	–	6^*	14^*
lmer	10	1	–	–	–	–	–

Table S3 The standard deviation and the mean squared error (separated by ;) of estimating β_0, β_1 and β_2 on a scale of 10^{-2}. All the methods produce the same results after rounding.

		m=50			m=100		
Parameter	n_c	$\rho = 0.05$	$\rho = 0.1$	$\rho = 0.5$	$\rho = 0.05$	$\rho = 0.1$	$\rho = 0.5$
β_0	10	7.32; 0.54	9.23; 0.85	17.85; 3.19	6.47; 0.42	8.44; 0.71	17.57; 3.09
	30	4.21; 0.18	5.19; 0.27	10.22; 1.05	3.84; 0.15	4.89; 0.24	9.97; 0.99
	50	3.27; 0.11	4.05; 0.16	7.67; 0.59	2.89; 0.08	3.71; 0.14	7.74; 0.60
	100	2.39; 0.06	2.84; 0.08	5.48; 0.30	2.04; 0.04	2.72; 0.07	5.58; 0.31
β_1	10	6.73; 0.45	6.66; 0.44	4.95; 0.24	4.86; 0.24	4.56; 0.21	3.50; 0.12
	30	3.93; 0.15	3.81; 0.14	2.90; 0.08	2.75; 0.08	2.66; 0.07	2.00; 0.04
	50	2.98; 0.09	2.92; 0.09	2.27; 0.05	2.18; 0.05	2.10; 0.04	1.56; 0.02
	100	2.25; 0.05	2.10; 0.04	1.57; 0.02	1.50; 0.02	1.47; 0.02	1.10; 0.01
β_2	10	3.49; 0.12	3.30; 0.11	2.42; 0.06	2.41; 0.06	2.39; 0.06	1.75; 0.03
	30	1.93; 0.04	1.90; 0.04	1.42; 0.02	1.43; 0.02	1.35; 0.02	0.99; 0.01
	50	1.50; 0.02	1.48; 0.02	1.11; 0.01	1.09; 0.01	1.03; 0.01	0.78; 0.01
	100	1.06; 0.01	1.06; 0.01	0.79; 0.01	0.75; 0.01	0.74; 0.01	0.55; < 0.1

Table S4 The standard deviation and the mean squared error (separated by ;) of estimating σ^2 on a scale of 10^{-2}. After rounding, results of "gls", "lme" and "lmer" are identical. Identical results of the "geese" and the "gls" methods are omitted.

		m=50			m=100		
n_c	Method	$\rho = 0.05$	$\rho = 0.1$	$\rho = 0.5$	$\rho = 0.05$	$\rho = 0.1$	$\rho = 0.5$
10	geese	4.01; 0.16	4.40; 0.20	13.37; 1.91	2.98; 0.09	3.62; 0.14	13.25; 1.85
	gls		4.41; 0.20	13.30; 1.90			13.24; 1.85
30	geese	2.28; 0.05	2.66; 0.07	7.84; 0.63	1.72; 0.03	2.17; 0.05	7.72; 0.61
	gls			7.86; 0.63			
50	geese	1.79; 0.03	2.06; 0.04	6.26; 0.39	1.31; 0.02	1.72; 0.03	6.01; 0.36
	gls			6.21; 0.39			5.99; 0.36
100	geese	1.27; 0.02	1.49; 0.02	4.27; 0.18	0.95; 0.01	1.20; 0.01	4.23; 0.18
	gls		1.48; 0.02	4.24; 0.18			4.22; 0.18

Table S5 The standard deviation and the mean squared error (separated by ;) of estimating ρ on a scale of 10^{-2}. After rounding, results of "gls", "lme" and "lmer" are identical. Identical results of the "geese" and the "gls" methods are omitted.

		m=50			m=100		
n_c	Method	$\rho = 0.05$	$\rho = 0.1$	$\rho = 0.5$	$\rho = 0.05$	$\rho = 0.1$	$\rho = 0.5$
10	geese	2.76; 0.08	4.59; 0.22	12.58; 1.88	2.47; 0.07	4.06; 0.18	11.90; 1.69
	gls		4.54; 0.22	12.20; 1.80	2.47; 0.06	4.04; 0.18	11.84; 1.67
30	geese	1.69; 0.03	2.76; 0.08	6.86; 0.50	1.43; 0.02	2.57; 0.07	6.71; 0.48
	gls	1.68; 0.03	2.73; 0.08	6.72; 0.48		2.56; 0.07	6.64; 0.47
50	geese	1.31; 0.02	2.18; 0.05	5.56; 0.32	1.12; 0.01	2.01; 0.04	5.19; 0.28
	gls	1.29; 0.02	2.14; 0.05	5.31; 0.29		2.00; 0.04	5.12; 0.27
100	geese	0.95; 0.01	1.56; 0.02	3.73; 0.14	0.78; 0.01	1.40; 0.02	3.61; 0.13
	gls	0.94; 0.01	1.53; 0.02	3.56; 0.13	0.77; 0.01		3.58; 0.13

Table S6 The standard deviation and the mean squared error (separated by ;) of estimating σ_u^2 and σ_ϵ^2 on a scale of 10^{-2}. After rounding, results of "gls", "lme" and "lmer" are identical.

		m=50			m=100		
Parameter	n_c	$\rho = 0.05$	$\rho = 0.1$	$\rho = 0.5$	$\rho = 0.05$	$\rho = 0.1$	$\rho = 0.5$
σ_u^2	10	1.73; 0.03	3.03; 0.10	13.20; 1.86	1.55; 0.03	2.68; 0.08	13.13; 1.82
	30	1.05; 0.01	1.80; 0.03	7.77; 0.61	0.90; 0.01	1.70; 0.03	7.70; 0.60
	50	0.81; 0.01	1.42; 0.02	6.20; 0.39	0.70; < 0.01	1.34; 0.02	5.97; 0.36
	100	0.59; < 0.01	1.01; 0.01	4.20; 0.18	0.49; < 0.01	0.93; 0.01	4.22; 0.18
σ_ϵ^2	10	3.70; 0.14	3.34; 0.11	1.89; 0.04	2.63; 0.07	2.45; 0.06	1.37; 0.02
	30	2.04; 0.04	1.99; 0.04	1.09; 0.01	1.50; 0.02	1.41; 0.02	0.78; 0.01
	50	1.63; 0.03	1.51; 0.02	0.84; 0.01	1.14; 0.01	1.06; 0.01	0.61; < 0.01
	100	1.15; 0.01	1.11; 0.01	0.61; < 0.01	0.81; 0.01	0.79; 0.01	0.44; < 0.01

Table S7 The coverage proportion (%) of β_0. The "gls-c" confidence intervals are obtained by the application of the "confint" function to a "gls" fitted object, and the "gls-i" confidence intervals are obtained by the application of the "intervals" function (Table 1). Specifying the "Wald" option of the "confint" functions yields the "lmer-w" confidence intervals and the option "profile" produces the "lmer-p" confidence intervals.

		m=50			m=100		
n_c	Method	$\rho = 0.05$	$\rho = 0.1$	$\rho = 0.5$	$\rho = 0.05$	$\rho = 0.1$	$\rho = 0.5$
10	geese	90.00	90.45	89.25	90.75	90.85	90.30
	gls-c	91.50	90.95	89.55	91.95	91.05	90.30
	gls-i	91.50	90.95	89.55	91.95	91.20	90.30
	lme	91.45	91.00	89.55	91.90	91.05	90.30
	lmer-w	91.35	91.00	89.50	91.90	91.00	90.20
	lmer-p	92.65	92.95	92.30	93.55	93.00	92.70
30	geese	93.90	93.55	93.60	93.10	93.35	92.80
	gls-c	94.30	94.15	93.65	93.35	93.55	93.05
	gls-i	94.30	94.20	93.65	93.35	93.55	93.05
	lme	94.30	94.15	93.65	93.35	93.55	93.05
	lmer-w	94.30	94.10	93.65	93.35	93.55	93.00
	lmer-p	94.50	94.90	94.45	94.05	94.20	93.65
50	geese	93.85	94.15	95.40	94.00	94.35	94.50
	gls-c	94.30	94.10	95.30	94.35	94.10	94.55
	gls-i	94.30	94.10	95.30	94.35	94.10	94.55
	lme	94.30	94.10	95.30	94.35	94.10	94.55
	lmer-w	94.30	94.10	95.30	94.35	94.10	94.55
	lmer-p	94.50	94.25	95.65	94.60	94.50	94.70
100	geese	93.65	94.75	94.80	94.90	94.15	94.05
	gls-c	93.80	95.00	94.75	95.10	94.15	94.05
	gls-i	93.80	95.00	94.75	95.10	94.15	94.05
	lme	93.80	95.00	94.75	95.10	94.13	94.06
	lmer-w	93.80	95.00	94.75	95.10	94.15	94.05
	lmer-p	93.95	95.30	95.05	95.30	94.35	94.45

Table S8 The coverage proportion (%) of β_1. The "gls-c" confidence intervals are obtained by the application of the "confint" function to a "gls" fitted object, and the "gls-i" confidence intervals are obtained by the application of the "intervals" function (Table 1). Specifying the "Wald" option of the "confint" functions yields the "lmer-w" confidence intervals and the option "profile" produces the "lmer-p" confidence intervals.

		m=50			m=100		
n_c	Method	$\rho = 0.05$	$\rho = 0.1$	$\rho = 0.5$	$\rho = 0.05$	$\rho = 0.1$	$\rho = 0.5$
10	geese	91.20	89.80	90.50	90.85	90.80	90.50
	gls-c	95.45	94.50	94.40	94.85	95.65	94.60
	gls-i	95.45	94.55	94.45	94.85	95.65	94.60
	lme	95.45	94.50	94.40	94.85	95.65	94.60
	lmer-w	95.40	94.50	94.40	94.80	95.65	94.55
	lmer-p	95.45	94.50	94.40	94.85	95.65	94.60
30	geese	93.20	93.80	93.65	92.90	94.15	94.15
	gls-c	94.80	95.30	95.30	94.50	95.15	95.35
	gls-i	94.90	95.30	95.35	94.50	95.15	95.35
	lme	94.80	95.30	95.30	94.50	95.15	95.35
	lmer-w	94.80	95.30	95.20	94.50	95.15	95.35
	lmer-p	94.80	95.30	95.30	94.50	95.15	95.35
50	geese	94.75	94.35	93.45	94.00	93.25	94.70
	gls-c	95.55	95.55	93.90	94.80	94.15	95.10
	gls-i	95.55	95.55	93.90	94.85	94.15	95.10
	lme	95.55	95.55	93.90	94.80	94.15	95.10
	lmer-w	95.55	95.55	93.90	94.80	94.15	95.05
	lmer-p	95.55	95.55	93.90	94.80	94.15	95.10
100	geese	93.45	94.65	94.70	94.80	94.80	94.20
	gls-c	93.95	95.30	95.35	95.30	95.60	94.55
	gls-i	93.95	95.30	95.35	95.30	95.60	94.55
	lme	93.95	95.30	95.35	95.30	95.59	94.66
	lmer-w	93.95	95.30	95.30	95.30	95.60	94.55
	lmer-p	93.95	95.30	95.35	95.30	95.60	94.55

Table S9 The coverage proportion (%) of β_2. The "gls-c" confidence intervals are obtained by the application of the "confint" function to a "gls" fitted object, and the "gls-i" confidence intervals are obtained by the application of the "intervals" function (Table 1). Specifying the "Wald" option of the "confint" functions yields the "lmer-w" confidence intervals and the option "profile" produces the "lmer-p" confidence intervals.

		m=50			m=100		
n_c	Method	$\rho = 0.05$	$\rho = 0.1$	$\rho = 0.5$	$\rho = 0.05$	$\rho = 0.1$	$\rho = 0.5$
10	geese	88.95	91.00	90.85	89.25	89.60	90.00
	gls-c	94.40	95.60	95.80	95.15	94.35	95.45
	gls-i	94.45	95.60	95.85	95.15	94.35	95.45
	lme	94.35	95.60	95.80	95.15	94.35	95.45
	lmer-w	94.30	95.55	95.70	95.10	94.35	95.40
	lmer-p	94.40	95.60	95.80	95.15	94.35	95.45
30	geese	94.05	94.45	93.80	92.95	93.60	93.25
	gls-c	95.00	95.55	95.45	94.10	94.85	95.05
	gls-i	95.00	95.55	95.45	94.10	94.85	95.05
	lme	95.00	95.55	95.45	94.10	94.80	95.05
	lmer-w	95.00	95.55	95.45	94.10	94.80	95.05
	lmer-p	95.00	95.55	95.45	94.10	94.80	95.05
50	geese	94.10	93.85	93.85	94.20	94.55	93.75
	gls-c	95.10	94.85	95.20	94.40	95.25	94.85
	gls-i	95.15	94.85	95.25	94.40	95.25	94.85
	lme	95.10	94.85	95.20	94.40	95.25	94.85
	lmer-w	95.10	94.85	95.20	94.40	95.25	94.85
	lmer-p	95.10	94.85	95.20	94.40	95.25	94.85
100	geese	95.00	94.50	94.65	95.15	94.50	94.80
	gls-c	95.75	94.75	94.90	95.60	95.00	95.10
	gls-i	95.75	94.75	94.90	95.60	95.00	95.10
	lme	95.75	94.75	94.90	95.60	95.04	95.12
	lmer-w	95.75	94.75	94.90	95.60	95.00	95.10
	lmer-p	95.75	94.75	94.90	95.60	95.00	95.10

Table S10 The coverage proportion (%) of σ^2 ("geese") or σ ("gls"), and ρ.

Parameter	n_c	Method	$m = 50$			$m = 100$		
			$\rho = 0.01$	$\rho = 0.1$	$\rho = 0.5$	$\rho = 0.01$	$\rho = 0.1$	$\rho = 0.5$
σ^2	10	geese	88.75	88.70	73.15	88.25	85.60	74.75
	30		93.35	92.10	88.05	93.25	91.15	86.85
	50		93.55	93.65	89.40	95.10	92.20	90.35
	100		94.15	93.35	94.00	94.20	93.70	92.15
σ	10	gls	94.45	94.30	82.31	93.20	91.80	82.79
	30		95.20	94.75	91.23	95.20	93.55	89.30
	50		94.60	95.15	92.05	96.15	93.50	91.29
	100		94.75	94.05	92.78	95.10	94.60	88.86
ρ	10	geese	76.75	75.35	75.95	73.35	75.40	78.70
		gls	91.70	90.45	88.80	88.15	90.35	88.91
	30	geese	86.95	86.20	90.30	87.95	86.90	88.95
		gls	93.80	92.35	93.85	94.30	92.75	92.28
	50	geese	90.75	90.45	90.30	90.85	89.50	91.40
		gls	94.85	93.55	93.21	94.60	93.50	91.96
	100	geese	91.90	91.40	94.05	92.80	92.45	93.05
		gls	94.40	94.00	94.10	95.05	93.95	89.88

Table S11 The coverage proportion (%) of σ_u and σ_ϵ.

Parameter	n_c	Method	$m = 50$			$m = 100$		
			$\rho = 0.01$	$\rho = 0.1$	$\rho = 0.5$	$\rho = 0.01$	$\rho = 0.1$	$\rho = 0.5$
σ_u	10	lme	99.24	98.60	88.65	99.25	92.40	89.50
		lmer	93.50	92.95	91.75	90.60	93.10	92.75
	30	lme	96.50	93.65	94.20	95.65	93.75	93.30
		lmer	94.30	93.40	94.85	95.15	94.25	94.20
	50	lme	96.20	93.95	93.70	95.05	93.60	94.15
		lmer	95.05	93.75	94.25	94.85	93.85	94.70
	100	lme	95.00	94.75	95.50	95.35	94.58	94.21
		lmer	94.70	94.65	95.65	95.30	95.00	94.85
σ_ϵ	10	lme	94.80	95.74	94.95	94.12	94.15	94.65
		lmer	94.75	95.95	95.25	94.10	94.35	94.50
	30	lme	95.45	95.10	95.30	94.40	95.30	94.65
		lmer	95.45	94.95	95.20	94.55	95.50	94.55
	50	lme	94.80	95.60	95.35	95.55	95.65	94.80
		lmer	94.80	95.60	95.30	95.70	95.55	94.85
	100	lme	95.00	94.10	94.50	95.05	93.98	94.21
		lmer	95.05	94.15	94.35	95.10	93.85	94.15
